# Variable Pringle Maneuvers and Effect on Intestinal Epithelium in Rats. A Pilot Experimental Study in Rats

**DOI:** 10.1371/journal.pone.0140707

**Published:** 2015-10-23

**Authors:** Dimitrios Dimitroulis, Demetrios Moris, Emmanouil Pikoulis, Eleftherios Spartalis, Georgios Kontadakis, Bart Vrugt, Serena Valsami, Gregory Kouraklis

**Affiliations:** 1 Second Department of Propedeutic Surgery, "Laikon" General Hospital, National and Kapodistrian University of Athens, Athens, Greece; 2 First Department of Surgery, "Laikon" General Hospital, National and Kapodistrian University of Athens, Athens, Greece; 3 Laboratory of Molecular Oncology, Clinic of Oncology, University Hospital Zürich, Zürich, Switzerland; University of Catania, ITALY

## Abstract

**Background:**

It is observed that combined liver and colon surgery especially when this includes major liver resection with Pringle maneuver (PM) performance does not have a favorable outcome. Aim of our experimental study is to investigate the impact of portal triad occlusion on the large bowel and intra-abdominal inflammation and potent protective effects of the variants of (PM) in the combined surgical cases.

**Materials and Methods:**

Forty-four rats were divided into four groups. In group A (control group), 1cm of the left partial colon was resected and then an end-to-end anastomosis was performed. In group B, a continuous PM for 30 minutes was performed followed by resection of 1cm of the left colon and an end-to-end anastomosis. In group C, the left colonic resection and anastomosis was performed after intermittent PM (IPM), which was 10 minutes PM followed by 5 minutes reperfusion repeated for three circles. In group D, an ischemic preconditioning for 10 minutes was initially performed followed by 5 minutes reperfusion and then continuous PM for 30 minutes. Finally the rats in group D underwent a 1cm left colonic resection and an end-to-end anastomosis.

**Results:**

The percentage of colitis was higher in the B group (P = 0,19). The percentage of inflammation was not significantly higher even when we compared all “occlusion” groups (B+C+D) with the sham group. No evidence of pancreatitis was found in the sham group whereas amylase and lipase levels were higher in Groups B, C and D together (P = 0,0267). The comparison of group A to group B showed a significant difference (P = 0,0014) caused by continuous PM for 30 minutes, but there was no such result after IPM.

**Conclusions:**

Major liver resections are performed with PM in order to minimize intra-operative blood loss. In the combined cases of colon surgery and major liver resections where PM is needed our results showed that IPM presents with better outcome and could be preferred compared with the other PM variants.

## Introduction

Colorectal cancer (CRC) remains an important public health problem as it is the third leading cause of death for both men and women in the United States and the most frequent form of cancer among patients aged 75 years and older[1]. Approximately 10–25% of patients with colorectal cancer present with colorectal liver metastases (CRLM) at the time of initial diagnosis [2]. The only therapeutic modality with intention to treat is surgical resection of both the primary and metastatic tumor in combination with chemo-radiotherapy. However, as not all patients are candidates for resection two major issues arise in this treatment strategy requiring a multidisciplinary approach. The first is the timing of chemotherapy and the second is the timing of liver resection. The liver surgeon has to decide whether to proceed to combined (liver and colon) or staged surgery and if he chooses a staged procedure which organ first, a matter which is controversial in the pertinent literature.

The advantage of a combined surgery is both the shortening of hospital stay and financial costs as well as less psychological stress for the patient while the staged procedure offers a minimization of the risks of two major operations [3]. However, the patient’ safety is the critical issue. Several studies have been published in the pertinent literature demonstrating that simultaneous liver and colon resection shows a higher mortality in comparison to staged resection [4–6]. On the contrary, there have been also some studies published revealing that a combined resection can be performed without any deaths [7–10]. Staged resection is recommended in the cases of chemotherapy performance between the two resections or in the case where an extended liver resection is performed [4, 5, 9, 10]. However, all these studies either represent the results of one center or are retrospective. Recent population studies, trying to resolve the problem of the timing of liver resection, conclude that colorectal resection with limited liver resection can be performed with similar mortality and morbidity to colectomy alone [5, 11, 12]. Major debate in the pertinent literature occurs regarding the timing of treatment of the metastatic disease in the case of synchronous colorectal liver metastases. This debate depicts the lack of standardized treatment for these complex cases. The national guidelines in the United States offer all treatment modalities with the same validity. The decision should be made for every single patient taking into consideration the extend of both colorectal and liver disease and the performance status of the patient. Our experimental study provides more technical and physiological details of a combined liver and colon procedure in order to reduce complications and advance surgical outcomes in the case of combined liver and colon resection [13, 14]. For patients, though, who require a lobectomy or more extended resections strong consideration should be given to a staged approach and each case should undergo an individualized strategy. Another surgical era where liver and colon surgery is combined is trauma. However, in this field we do not have the opportunity to discuss which organ should be operated first as the situation is very urgent during an explorative laparotomy.

On the other hand technical innovations focusing on minimizing bleeding during liver parenchymal transection and the need for blood transfusion have increased worldwide liver resections both for primary as for secondary liver tumors as an intention to treat strategy. Portal triad clamping (Pringle Maneuver, PM) has been used since the early 20^th^ century to reduce bleeding during liver parenchyma transaction [15, 16]. However, the PM causes ischemic injury to the remaining liver increasing the risk for postoperative complications and liver failure. For this reason several variations of the PM have been introduced in order to minimize the period of liver ischemia decreasing thus the reperfusion injury. These variations include ischemic preconditioning, intermittent clamping and recently pharmacological preconditioning. Intermittent clamping consists of repeated circles of clamping while between them short periods of reperfusion are performed [17, 18] while ischemic preconditioning represents a limited sequence of ischemia and reperfusion followed by a prolonged ischemic period [19]. Pharmacological preconditioning is a relative new approach in the setting of reducing blood loss and concomitant liver protection during liver parenchymal transaction using volatile anesthetic agents [20].

However, almost all of the experimental and clinical studies published in the literature focus on the impact of portal triad occlusion on the liver through ischemia—reperfusion injury and all variants of Pringle maneuver were performed in order to minimize liver parenchyma damage while the impact of this manipulation on the bowel is of minimal interest.

The “gut-liver axis” closely links gut function and liver function in health and disease. Under disease situation, intestinal microbiota imbalance can aggravate liver injury and promote chronic inflammatory disease of the liver [21]. Meanwhile, hepatic injury or disease, such as nonalcoholic steatohepatitis, alcoholic steatohepatitis, and cirrhosis, always follows changes in intestinal permeability and microbial composition [21].

Our clinical observation that combined liver and colon surgery especially when this includes major liver resection with Pringle maneuver performance does not have a favorable outcome for the patient has urged us to perform an experimental study in order to investigate the impact of portal triad occlusion on the large bowel and intra-abdominal inflammation process and potent protective effects of the variants of Pringle maneuver.

### General Principles

Ischemia reperfusion injury (IRI) is a pathophysiologic process where hypoxic organ damage is accentuated by following restoration of blood flow and oxygen delivery to the ischemically-damaged tissue. The latter results to liver exposure not only to direct cellular damage from ischemic insult, but also to delayed dysfunction and tissue injuries resulting from activation of inflammatory cascades after reperfusion [22]. Hepatic IRI is often encountered in various clinical situations including liver transplantation, trauma, shock state followed by resuscitation and elective liver resections with inflow occlusion that is frequently used to minimize blood loss.

Ischemic preconditioning (IPC) refers to a strategy in which prior transient ischemia induces a state of protection against subsequent prolonged damage [23]. In hepatobiliary surgery, Pringle’s maneuver, represents a typical paradigm of an effective strategy to reduce blood loss and transfusion requirements. Although the exact protective mechanisms are unknown, IPC is thought to result in adenosine and nitric oxide release, which protects the liver against subsequent prolonged episodes of continuous ischemia [24].

However, noble Pringle’s maneuver easily results in adverse effects, especially on marginal livers such as cirrhosis and steatosis, because a safe ischemic period for normal liver might be conversely crucial for marginal livers to be yielded to liver failure [25]. Same difficulties are in ischemic insults for preconditioning. To date, there has been several randomized, controlled clinical trials evaluating the efficacy of IPC in liver resections, however, most of which failed to support clinical benefits of IPC despite various protective results from numerous experimental settings [26–28].

## Materials and Methods

All experiments were performed on male Wistar rats and all procedures were performed in accordance with the Athens University Institutional Animal Care Guidelines and were approved by the local Animal Ethics Committee. This protocol was approved by the General Directorate of Veterinary Services, according to Greek legislation regarding ethical and experimental procedures (Presidential Decree 160/1991, in compliance with the EEC Directive 86/609 and Law 2015/1992 and in conformance with the European Convention ‘for the protection of vertebrate animals used for experimental or other scientific purposes’, 123/1986). For the study design forty four rats were divided into four groups (Table 1). In group A (control group) containing eleven rats (mean weight 280.82gr) 1cm of the left partial colon was resected and then an end-to-end anastomosis was performed with continuous suture Prolene No 5/0 without any manipulation of the portal triad. In group B containing eleven (mean weight 344.5gr) rats a continuous portal triad occlusion (continuous PM) for 30 minutes was performed followed by resection of 1cm of the left colon and an end-to-end anastomosis similar to the control group. In group C with eleven rats (mean weight 325.60gr) the left colonic resection and anastomosis was performed after intermittent portal triad occlusion (intermittent PM), which is described as portal triad occlusion for 10 minutes followed by 5 minutes reperfusion repeated for three cycles. In the end in group D also containing eleven (mean weight 322.54) rats an ischemic preconditioning for 10 minutes was initially performed followed by 5 minutes reperfusion and then continuous portal triad occlusion for 30 minutes. Finally the rats in group D underwent a 1cm left colonic resection and an end-to-end anastomosis similar to the other groups. At this point it is important to mention that all manipulations on the portal triad were performed prior to the left colonic resection.

**Table 1:** Parameters of the experiment.

*n*	IN	G	S	DoE	PM	C	CG	P	PG
1	1	A	M	5		*	1	*	1
2	2	A	M	5			0	*	1
3	3	A	M	5		*	1		0
4	4	A	M	5			0		0
5	5	A	M	5			0	*	1
6	6	A	M	5		*	1	*	1
7	7	A	M	5			0		0
8	8	A	M	5			0		0
9	9	A	M	5		*	2	*	1
10	10	A	M	5			0	*	1
11	11	A	M	5			0	*	1
12	1	B	M	5	30 min		0	*	1
13	2	B	M	5	30 min		0	*	1
14	3	B	M	5	30 min	*	3	*	3
15	4	B	M	5	30 min	*	3	*	3
16	5	B	M	5	30 min	*	3	*	3
17	6	B	M	5	30 min	*	2	*	2
18	7	B	M	5	30 min		0	*	2
19	8	B	M	5	30 min	*	3	*	1
20	9	B	M	5	30 min	*	3	*	3
21	10	B	M	5	30 min	*	1	*	1
22	11	B	M	5	30 min	*	2	*	2
23	1	C	M	5	10 min IPM x3		0	*	1
24	2	C	M	5	10 min IPM x3	*	2	*	1
25	3	C	M	5	10 min IPM x3	*	2		0
26	4	C	M	5	10 min IPM x3		0		0
27	5	C	M	5	10 min IPM x3	*	3	*	3
28	6	C	M	5	10 min IPM x3		0	*	2
29	7	C	M	5	10 min IPM x3	*	2	*	1
30	8	C	M	5	10 min IPM x3	*	2	*	1
31	9	C	M	5	10 min IPM x3		0	*	1
32	10	C	M	5	10 min IPM x3		0	*	2
33	11	C	M	5	10 min IPM x3	*	2	*	2
34	1	D	M	5	10 min pC + 30 min PM		0	*	2
35	2	D	M	5	10 min pC + 30 min PM	*	3	*	3
36	3	D	M	5	10 min pC + 30 min PM		0	*	1
37	4	D	M	5	10 min pC + 30 min PM	*	3	*	2
38	5	D	M	5	10 min pC + 30 min PM	*	2	*	2
39	6	D	M	5	10 min pC + 30 min PM		0	*	3
40	7	D	M	5	10 min pC + 30 min PM	*	2	*	2
41	8	D	M	5	10 min pC + 30 min PM	*	3	*	3
42	9	D	M	5	10 min pC + 30 min PM	*	2	*	2
43	10	D	M	5	10 min pC + 30 min PM	*	2	*	2
44	11	D	M	5	10 min pC + 30 min PM		0	*	1

N: animal number; IN: postoperative identification number; G: group of animals; S: sex; PM: type of Pringle maneuver; IPM: intermittent Pringle maneuver; pC: pre-conditioning; DoE: date of euthanasia; C: colitis; CG: grade of colonic inflammation; P: peritonitis; PG: grade of peritoneal irritation,

* no footnotes are needed for this symbol.

Rats were anesthetized by isoflurane inhalation (Pittman-Moore, Chicago, Illinois, USA). All procedures were performed by a midline laparotomy, the portal triad was recognized and occluded with the use of a fine vascular clamp. After the performance of the colonic resection and the anastomosis as described above, the abdominal cavity was closed and these were allowed to wake-up. No antibiotics were given. All rats were also allowed standard food and water ad libitum. On postoperative day five all animals were sacrificed under general anesthesia, the abdomen was opened via the old laparotomy, an abdominal exploration was performed and a part of the left colon including the anastomosis as well as the left liver were resected and embedded in paraffin blocks.

The resected specimens were sent to the Laboratory of Pathology. Histological assessment of the slides was performed by two independent, blinded to the treatment modalities, pathologists. The tissue samples were processed in buffered formaldehyde during 24 h and embedded in paraffin. Multiple (3–4) transverse sections were taken from the small intestines and colon. In addition representative sections were taken from the pancreas and liver. From the different organ systems 4 μm sections were cut and stained routinely with hematoxylin-eosin (HE) and elastic von Gieson (EvG). Histological assessment of the slides was performed by one independent pathologist, blinded to the treatment modalities. The histopathological changes included colitis and peritonitis, which were semi-quantitatively analyzed, based on the extension of the acute inflammatory process: 1+ = acute focal colitis; 2+ = acute segmental colitis with peritonitis and 3+ diffuse acute colitis with extensive peritonitis. S1 File contains data being analyzed in our study.

The percentages in each group of animals were compared using the Fisher’s exact test. The distribution of grading of each inflammatory condition among groups was compared with the Kruskal-Wallis test. In case of statistical significance, post-hoc analysis with the Dunn-Bonferroni method was employed to elaborate the specific pairs of groups with different distribution. All results used with a p level ≤ 0.05 were considered statistically significant. Statistical calculations were performed using the SPSS for Windows version 19.0 (SPSS Inc., Chicago, IL, U.S.A.).

## Results

### Colitis

**Fig 1:**
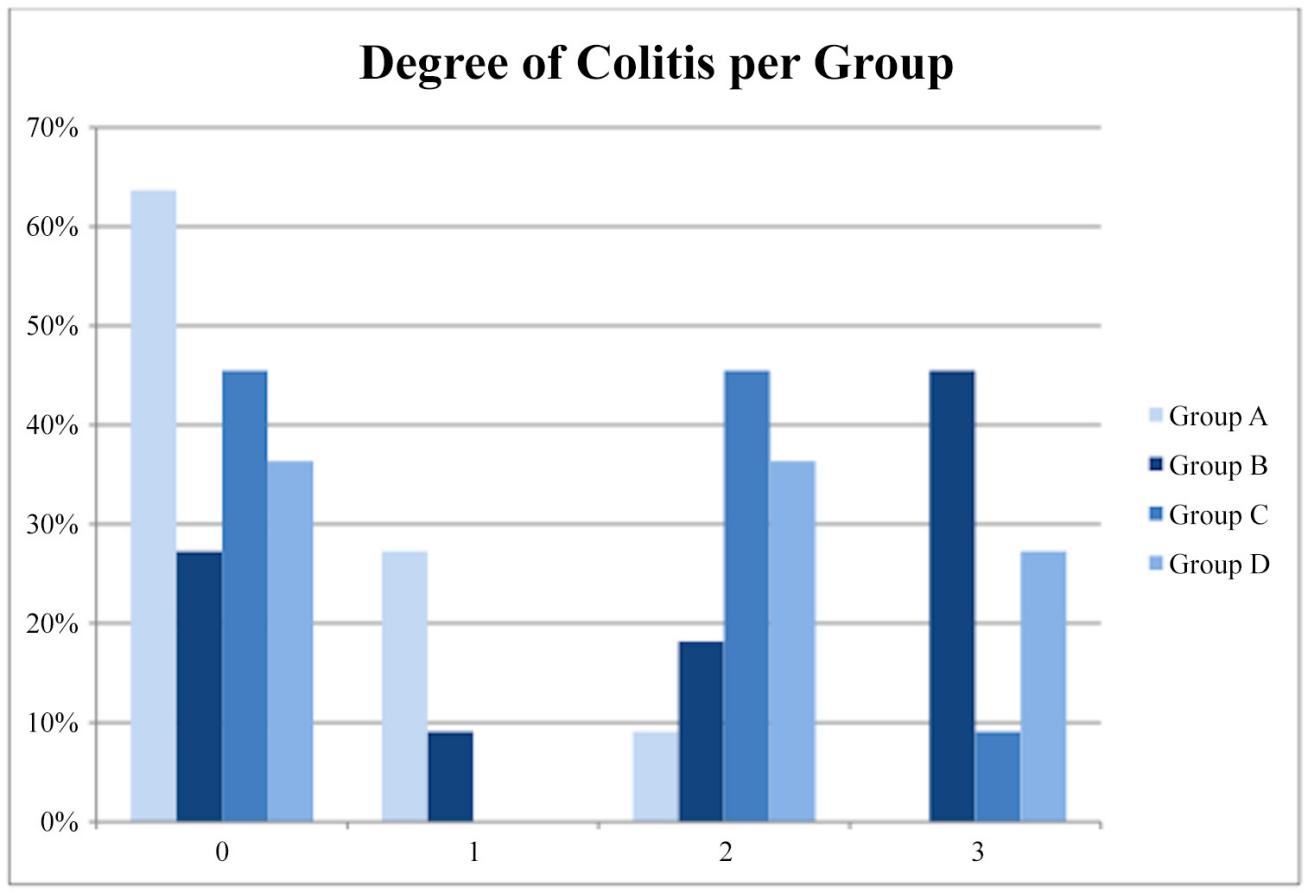
Distribution of the grades of colitis among the four groups.

**Fig 2:**
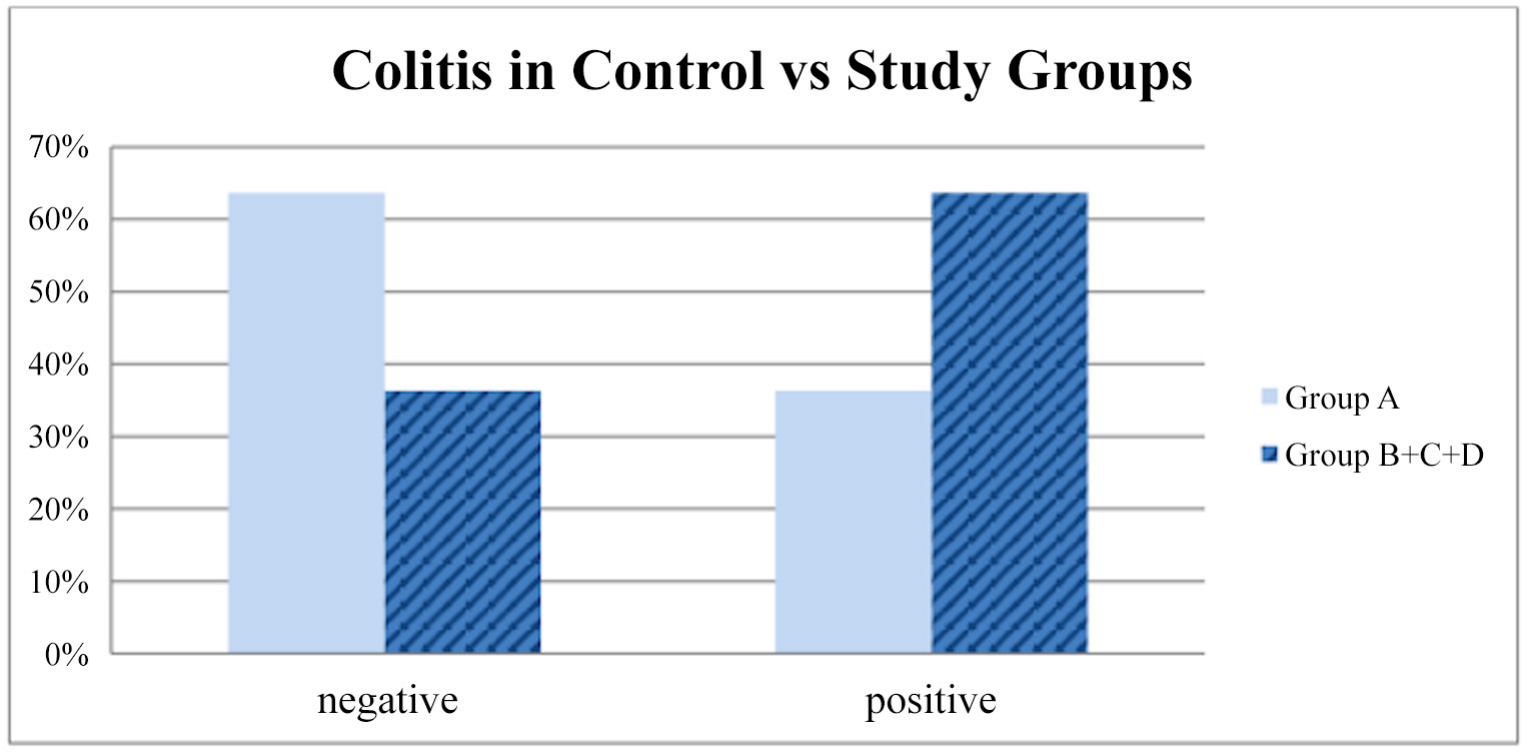
The percentage of colonic inflammation in the control group (A) compared to all “occlusion” groups (B+C+D) together (P = 0,16).

**Fig 3:**
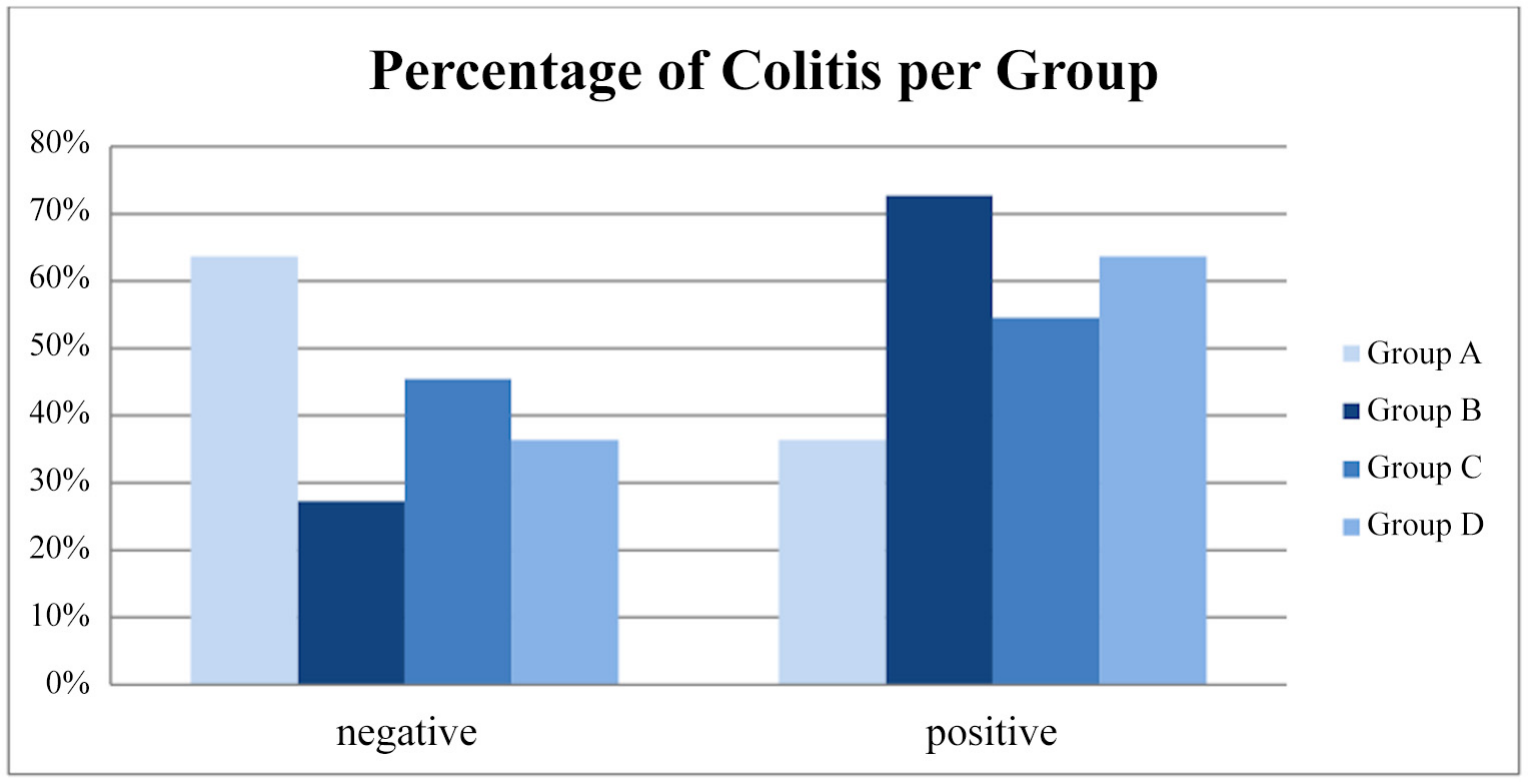
Percentage of specimens positive for colitis among all groups.

The percentage of colitis was higher in the B group of animals, especially grade 3 as evaluated by pathology (Table 2). This observation, however, was not confirmed by statistical analysis when comparing percentage or colitis- positive specimens between group B and control group (P = 0,19). The difference in distribution of colitis between groups did not reach statistical significance according to the Kruskal-Wallis test (p = 0,60). The distribution of the inflammatory findings of colitis is shown in Fig 1. The percentage of inflammation was not significantly higher even when we compared all “occlusion” groups (B+C+D) with the group A (P = 0,16) (Fig 2). Percentage of specimens positive for colitis was not higher in neither study group as compared to control group (P = 0,19, P = 0,6 and P = 0,3 respectively) (Fig 3).

**Table 2:** Distribution of the grades of colitis and peritonitis among the four groups.

**Colitis**	**G**	**MW (gr)**	**C**	**P (%)**	**MG**	**T**
	A	280.82	4	36,3	0.45	11
	B	344.5	8	72,7	1.82	11
	C	325.6	6	54,5	1.18	11
	D	322.54	7	63,6	1.55	11
Total			25	56.8	1.25	44
**Peritonitis**	**G**	**MW (gr)**	**P**	**P (%)**	**MG**	**T**
	A	280.82	7	63,6	0.64	11
	B	344.5	11	100	2	11
	C	325.6	9	81,8	1.27	11
	D	322.54	11	100	2.09	11
Total			38	86.4	1.5	44

### Peritonitis

**Fig 4:**
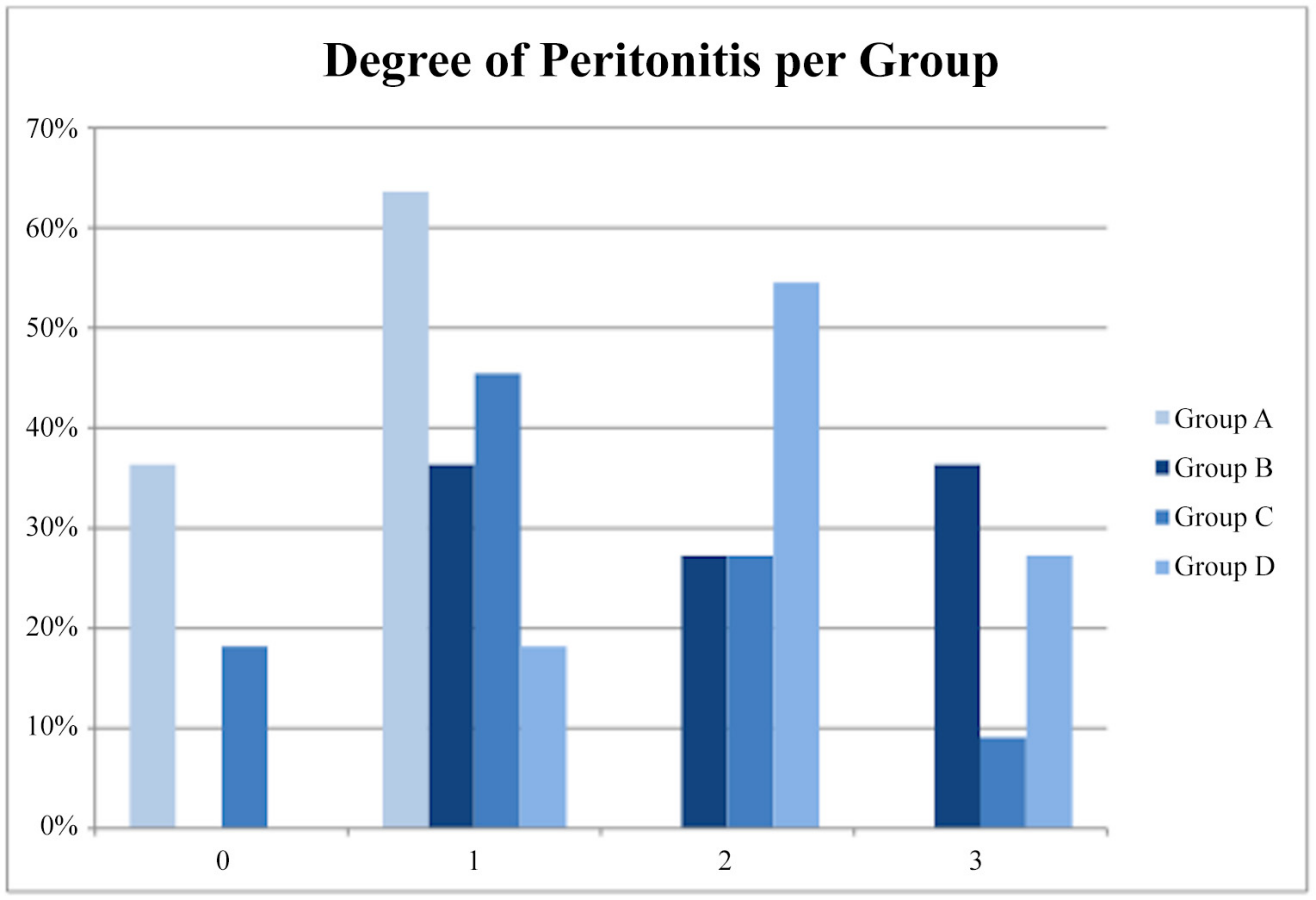
Distribution of the grades of peritonitis among the four groups.

**Fig 5:**
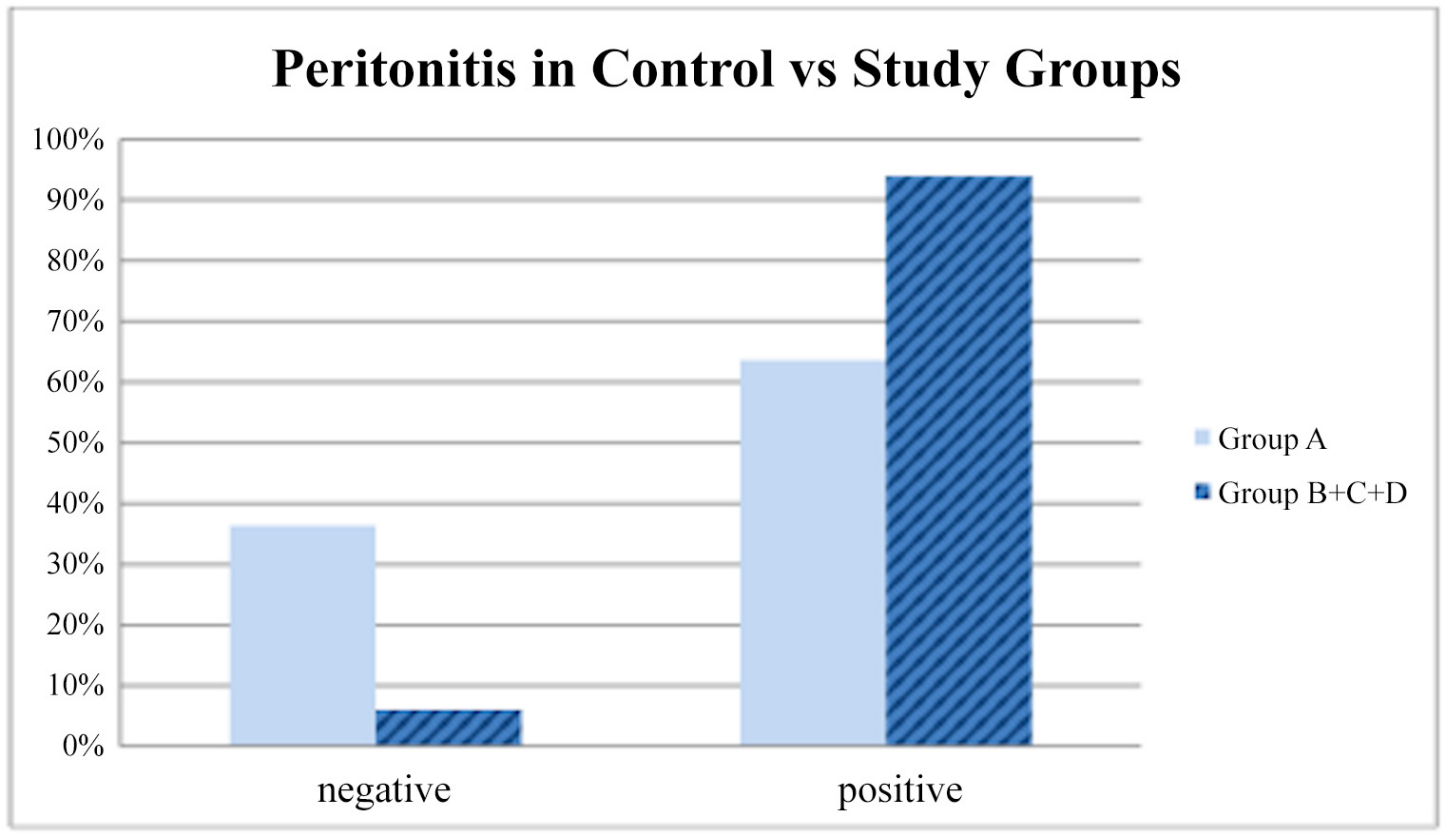
The percentage of peritoneal irritation in the sham group compared to all the other three groups together (P = 0,0269).

**Fig 6:**
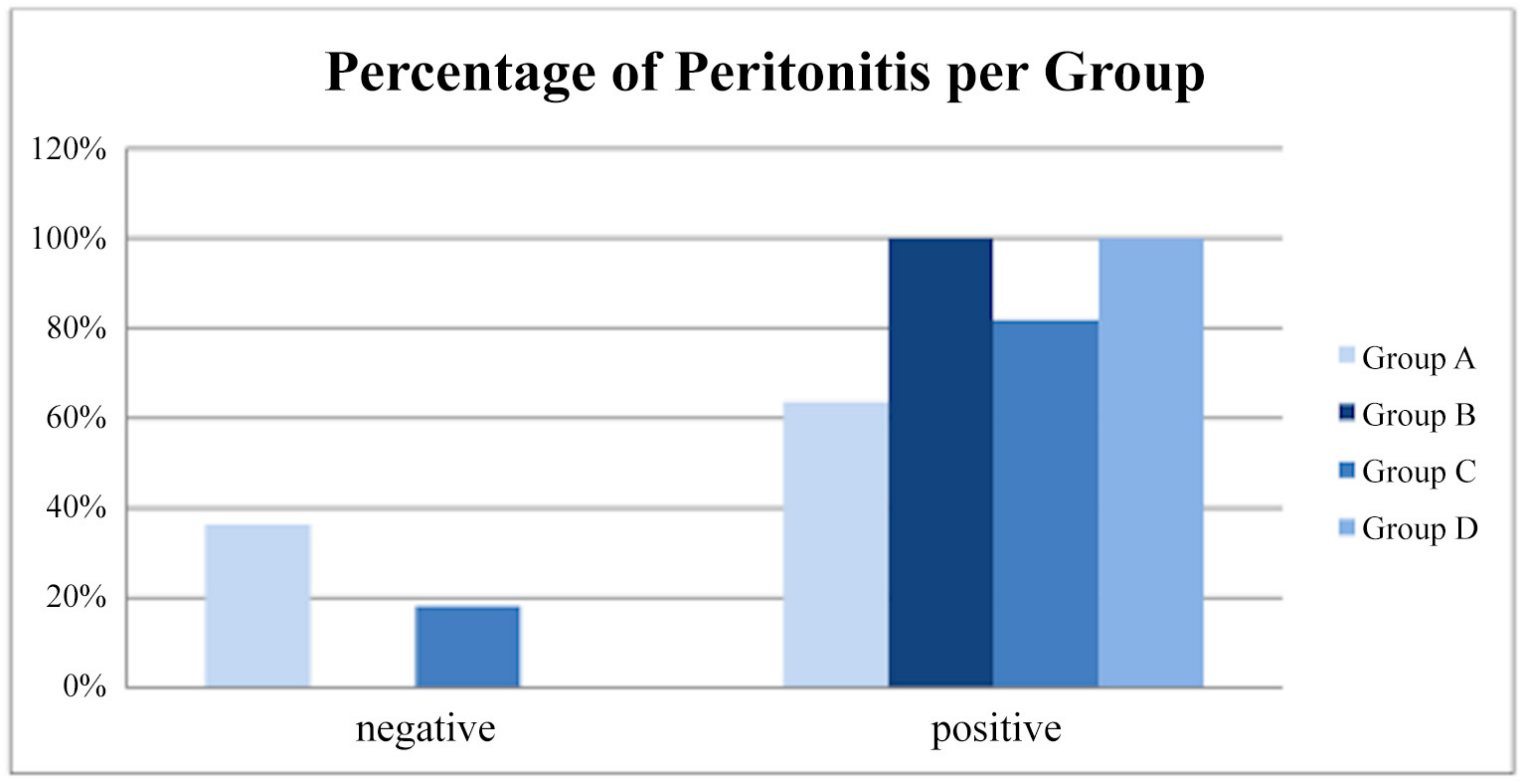
Percentage of specimens positive for peritonitis among all groups.

Grade 0,5 and Grade 1 peritonitis was found mostly in Group A. Grade 2 inflammation was higher in the occlusion groups B, C and D as shown in Fig 4. In accordance to that, the distribution of grade was found to have a statistically significant difference between groups (p = 0,01, Kruskal-Wallis test). Post-hoc analysis revealed that there was significant difference in distribution of grade of peritonitis between group A and group B and also between group A and group D (p = 0,04 and p = 0,01 respectively, Dunn-Bonferroni post-hoc test). Consequently, more severe peritonitis was encountered in groups B and D (Table 2). With respect to percentage of positive samples in each group, the comparison of the sham group with all the other three groups together was statistically significant (P = 0,0269) (Fig 5). This means that some form of peritoneal inflammation is caused after any occlusion maneuver. No significant differences, however, were obtained neither when we compared the four groups separately, nor when we compared the animal groups with each other. Detailed findings are shown in Fig 6.

### Pancreatitis

No evidence of pancreatitis was found in the sham group of animals. On the other hand, each one of the occlusion maneuvers induced various levels of pancreatic irritation attributed to the secretion of pancreatic enzymes and other mechanisms extensively described in the discussion section. Amylase and lipase levels were higher in Groups B, C and D together, compared to the sham group (A), indicating that portal triad occlusion (either continuous or intermittent) lead to a statistically significant percentage of pancreatitis (P = 0,0267). The comparison of group A to group B showed a significant difference (P = 0,0014) caused by continuous Pringle maneuver for 30 minutes, but there was no such result after intermittent occlusion (P = 0,4375 and P = 0,1099 respectively) (Table 3). This fact indicates an important issue regarding duration of occlusion and its correlation to pancreatic inflammation. Despite the difference in percentage of positive results between groups, according to the Kruskal-Wallis test the difference on the distribution of the grade of pancreatitis between groups was not statistically significant (p = 0.134).

**Table 3:** Distribution of the grades of pancreatitis among the four groups.

Pancreatitis	G	MW (gr)	n	P (%)	MG	T
	A	280,82	0	63,6	0	11
	B	344,5	4	100	2	11
	C	325,6	1	81,8	2	11
	D	322,54	2	100	2	11
Total			7	86,4	1	44

G: group of animals; MW: mean weight (gr); n: number of positive samples P: percentage of positive postoperative findings (%); MG: median grade of inflammation; T: total

## Discussion

Intra-operative blood loss and red blood cell transfusions are associated with short- and long-term complications in liver surgery, such as operative mortality or major complications that require post-operative radiologic or surgical intervention [29, 30] and also predisposing to post-resectional liver failure [30]. Furthermore, blood loss that requires blood transfusion has a negative impact on the overall postoperative oncological result [31]. For this purpose, several techniques have been implied in order to minimize blood loss during hepatic parenchymal transection. In recent years combined liver and colorectal surgery for CRLM and trauma has emerged. The effect on the intestine is not well studied.

As we have already mentioned, intermittent PM (IPM) may have a negative effect on outcome after liver resection as a consequence of liver damage due to ischemia-reperfusion injury (IRI) [25, 28, 32]. Petrowsky et al [33] showed that both ischemic preconditioning (IPC) and IPM appear to be equally effective in protecting against postoperative liver injury in non-cirrhotic patients undergoing major liver resection. However, IPM is associated with lower blood loss and shorter transection time.

IRI damage of the liver as a consequence of IPM has been well studied, however little is known about the effects of IPM on the gut. Clamping of the hepatoduodenal ligament leads to stasis in the portal vein and the superior and inferior mesenteric veins, thereby reducing splanchnic outflow [34], which in turn leads to intestinal hypoperfusion. The latter eventually results in enterocyte damage and gut-barrier loss [34]. Loss of intestinal epithelial integrity is clinically important, as it is associated with the development of sepsis and multiple organ failure following major surgery, trauma and shock [35].

The intimate anatomical and functional relationship between the intestine and the liver closely links the two organs in health and disease. Hepatic injury or disease generally follows changes in intestinal permeability and microbial composition. Ren et al [36] recently showed that liver IPC has protective effects on hepatic graft and beneficial roles on intestinal barrier function. On the other hand, they also found that gut IPC had little benefit on intestinal barrier function and microbial stability in a liver transplantation model except that gut IPC mildly attenuated intestinal mucosal epithelium integrity [36]. The possible reason was that gut IPC only mildly increased the tolerance of intestinal epithelial cells to IRI, but had little influence on hepatic graft, thus it could not prevent microbial disturbances and cascade inflammation induced by the injured graft function [36].

As in our study, the effect of splanchnic hypoperfusion on intestinal damage as a consequence of PM, intermittent or continuous, has been proven in some animal studies [37]. Sheen-Chen et al [38] showed recently in rats that occlusion of the hepatoduodenal ligament significantly increased jejunal apoptosis. Furthermore, Ochiai et al [39] showed that IPM caused intestinal epithelial cell damage and increased small intestinal permeability in rats.

On the other hand, data on the effect of IPM on the gut in man still are scarce. Dello et al [40] investigated the effect of IPM in human gut by performing PM in cycles of 15 and 30 minutes as well as in cycles of 15 minutes of selective hepatic occlusion (clamping of the right portal pedicle). The 15-IPM and 30-IPM groups were subsequently pooled and compared with controls (no-IPM) since there was no significant difference in intestinal epithelial cell damage between 15-IPM and 30-IPM [40]. On the contrary, a significant difference in intestinal epithelial cell damage between the total-IPM group, and the no-IPM group was found (p = 0.01) [40]. Moreover, there were no significant differences at any time point between selective IPM and no-IPM [40].

It remains unknown whether it is only the impaired microcirculation in the intestine during portal clamping which causes cell damage or that the temporal acute rise of venous pressure in the mesenteric system in itself also plays a role. King et al [41] showed that when splanchnic venous outflow was occluded in patients, both intestinal oxygen extraction ratio and portal venous lactate increased. An acute rise of venous pressure in the microcirculation in itself will probably also impair perfusion and consequently cause tissue hypoxia. It is most likely a combination of the two mechanisms that explains intestinal epithelial damage in these patients.

Beyond intestinal damage, it also seems important to evaluate the interesting finding of indirect pancreatic stimulation and inflammation after PM. The latter may occur when factors involved in maintaining cellular homeostasis become unbalanced. The initiating event could be anything which injures the acinar cell and impairs the secretion of zymogen granules.

The mechanisms leading to destructive effect on pancreatic acinar cells are unknown, but once a cellular injury pattern has been initiated, activated neutrophils exacerbate the problem by releasing superoxide (the respiratory burst) or proteolytic enzymes (cathepsins B, D, and G, collagenase and elastase)[42]. Finally, the release of cytokines, tumor necrosis factor-alpha (TNF-alpha), interleukin-6 (IL-6), and interleukin-8 (IL-8) by macrophages further mediates the local and/or systemic inflammatory responses [42]. The pro-inflammatory cytokines, especially TNF and IL-1, play a critical role throughout the inflammatory period [42].

In addition, acute pancreatitis can begin in the postoperative period of various types of surgery, including hepatic surgery [42]. Some reports have addressed the hyperamylasemia and pancreatitis states present after hepatic surgery and linked those to chronic liver disease or a prolonged PM [42, 43]. It has also been proven that PM can induce pancreatic venous congestion, hyperamylasemia and pancreatitis occurring secondarily to total clamping of the pancreatic venous system [44].

Aydede et al [45] pointed out that portal bed congestion can trigger an inflammatory response in the pancreas, with a directly proportional relationship between congestion and the changes. Unalp et al [42] showed that elevated levels of TNF-alpha were found only during PM, leading to an indirect relationship between PM and the TNF-alpha level.

There have also been some reports about hyperamylasemia secondary to portal congestion or PM during hepatic resection [43, 46]. However, PM was not found to have a direct effect on amylase levels [42].

All these information that are taken from our study and the relative literature on the subject should be evaluated in the frame whether performing IPM is favorable in patients undergoing liver surgery, such as in cases of colorectal metastatic liver disease. Hepatectomies without IPM can be performed safely due to advances in liver surgery such as the development of modern hemostatic devices and improvements in anesthesiological management [47]. Acute major bleeding remains an indication for IPM, but current evidence shows no benefit for IPM on outcome after liver resection [48]. Therefore in the modern era of liver surgery systematic use of IPM has become more often a subject of debate. It is already highlighted that total-IPM is associated with intestinal epithelial cell damage and endotoxemia [40]. This could facilitate infective postoperative complications as well as unsound enteric anastomoses due to the potential ischemic environment after PM. Unravelling this mechanism could help to (preoperatively) identify patients with a higher risk of these complications.

A possibly more safe approach than total IPM is selective IPM, which is performed by selectively excluding the right or left hemi-liver from the circulation. Selectively clamping the right or left portal pedicle is safe and feasible for patients with normal liver parenchyma and especially in cirrhotic patients the selective IPM induces less ischemic liver injury compared to total IPM [49]. One would expect that also the intestinal epithelial cell integrity is less compromised in these patients because splanchnic outflow is only partly reduced. This hypothesis was also confirmed by Dello et al [40].

## Conclusions

Major liver resections are performed with PM in order to minimize intra-operative blood loss. In the case of the combined colon surgery and major liver resection where PM is needed more often for colorectal liver metastases and less often in the case of major abdominal trauma, our results showed that IPM presents with better outcomes and could be preferred compared to the other variants.

As far as pancreatic inflammation is concerned, we showed that PM has an indirect correlation with pancreatic inflammation whereas IPM presents with less incidence of acute pancreatitis. Despite advances in experimental studies on the pancreas, the mechanism involved in pancreatic changes resulting in an acute increase of portal pressure secondary to portal venous clamping has not been fully clarified.

All in all, in this pilot study, it is shown that intermittent ischemia could protect hepatocytes from ischemia/reperfusion injury, but also show a therapeutic influence on an ectopic organ like the colon. This could appear to have very real clinical implications for surgeons performing colectomies in which hepatic metastases needed to be simultaneously resected.

## Supporting Information

S1 File Data analyzed in our study. (RAR)

## Abbreviations

(CRC): Colorectal cancer
(CRLM): colorectal liver metastases
(EvG): elastic von Giesson
(HE): hematoxylin-esoin
(IL-1): interleukin-1
(IL-6): interleukin-6
(IL-8): interleukin-8
(IPM): Intermittent Pringle Maneuver
(IRI): Ischemia reperfusion injury
(IPC): Ischemic preconditioning
(PM): Pringle maneuver
(TNF-alpha): tumor necrosis factor-alpha
